# Two Memoirs on the Variation of Color in the Venous Blood

**Published:** 1859-06

**Authors:** Claude Bernard


### TRANSLATIONS FROM FOREIGN JOURNALS.

[From Brown-Sequard's "Journal de la Physiologie."]

### TWO MEMOIRS ON THE VARIATIONS OF COLOR OF THE VENOUS BLOOD.

BY PROFESSOR CLAUDE BERNARD.

[Read to the Academy of Sciences, Aug. 9 and Sept. 6, 1858.]

TRANSLATED BY THOMAS BEVAN.

Continued from page 285.

V. It is very easy to demonstrate experimentally that, of the two nerves we have mentioned in the submaxillary gland, the one dilates the vessels, while the other contracts them.

The tympanico-lingual nerve enlarges the capillary vessels of the gland, and this enlargement is such, that when the nervous action is intense, the blood passes from the artery into the vein without losing the cardiac impulsion, and it is seen to flow from the vein of the gland with an intermittent jet, as if flowing from a real artery; afterwards, this venous pulsation disappears, from the moment the action of the tympanico-lingual nerve diminishes or completely ceases.

The sympathetic nerve, on the contrary, contracts and restricts the glandular blood-vessels in the most marked manner. When this nerve is excited, the constricted vessels allow less and less blood to pass. The sanguine fluid retained in the capillary vessels of the gland, flows feebly by the vein, exhibiting a dark color, and darker in proportion as the current is feebler.* When it happens sometimes, that the flow of blood has been suspended by the nervous influence, one sees, when this ceases to act, a quantity of very black blood escape first; afterwards the blood takes its red color, little by little, in proportion as its circulation is accelerated, and the blood, which had been previously retained in the tissue of the gland, is found to be expelled.

In the last analysis, we see that the two nerves, which modify

* When the vein is compressed or obstructed by a clot, this accidental cause also brings about a dark coloration of the blood. It is important to know these circumstances to guard against all the causes of error in the appreciation of the nervous influences.

the color of the blood red and dark, are two motor nerves, which act primitively by constricting or dilating the blood-vessels. The sympathetic nerve is the one which contracts the vessels; the tympanico-lingual the dilatator.*

VI. In the physiological condition of the submaxillary gland, —that is to say, in its normal functional state—we ought to represent to ourselves two orders of nerves as being constantly in activity and antagonism; so that the effective nervous action is always due to the nerve actually preponderating, and that the special influence of one of the two glandular nerves does not seem to manifest itself until it has previously annihilated the action of the other. What proves it is, that each of the nerves becomes more excitable, and reacts with more intensity, under the same excitant, when its antagonist nerve has been previously destroyed. This last phenomenon is very clear, especially for the tympanico-lingual. When this nerve, remaining intact, for example, we cut all the glandular filaments of the sympathetic, and afterwards place a little vinegar on the tongue, we see the ruddy blood flow from the vein with much greater rapidity, and the pulsations become much more energetic than in the normal condition of nervous antagonism,—that it to say, when the sympathetic has not been cut.

This difference in the excitability of the tympanico-lingual nerve is much the more interesting to establish, as it is found here measured by its normal physiological excitant, the gustative impression. All this shows us the existence of a sort of unstable equilibrium in the submaxillary gland, or, of a sort of functional balancing, incessant and determined, by the antagonism of the dilatator nerve and the constrictor of capillary vessels.†

* This is not the place to seek to give the explanation, which may be given in the present state of the science, of the enlargement of the vessels and the increase of activity of the glandular circulation under the nervous influence. I will limit myself here to the statement of the fact which appears to me important, and of which there is besides the utmost evidence.

† It may be said, as a general proposition, that in the physiological condition, the expulsion of the saliva from the gland corresponds with the activity of the tympanico-lingual nerve, and the repose of the same gland with the activity of the great sympathetic. The excitation of these two orders of nerves may always make the saliva flow; only the excitation of the tympanico-lingual causes a

The extreme dilatation of the capillary system coincides with the direct passage in the vein of the red pulsatile blood. The extreme contraction coincides with a feeble flow of blood, and with a dark color. Between these two extremes we can conceive all the intermediates, and observation may show them to us in experiments.

VII. To recapitulate: after having analyzed successively all the mechanical conditions by which the tympanico-lingual and great sympathetic cause the venous blood of the maxillary gland to appear, alternately, red and black, we have arrived at this conclusion: that these two nerves do not, in reality, act, but as agents of contraction and dilatation of the blood-vessels. This action, which differs in no way from the action of the motor nerves in general upon contractile or muscular elements, brings about, however, its results, by a chain of entirely natural phenomena, a series of physico-chemical modifications in the sanguineous fluid. When the sympathetic nerve constrictor of the vessels acts, the contact between the blood and the elements of the gland is prolonged, the chemical phenomena which result from the organic exchange which passes between the blood and the tissues has had time to operate, and the venous blood flows very dark. When, on the contrary, the tympanico-lingual nerve, which dilates the vessels, acts, the passage of the blood in the gland is rendered very rapid, the modifications of *veinosity*, which pass at the contact of the blood and the tissues, is otherwise accomplished, and the blood escapes from the vein with a ruddy tint, and presenting its aspect of arterial blood.

Thus, we may always seize between the primitive physiological action of the nerve, and the chemical phenomenon which follows, an intermediary, which modifies mechanically the special circulation of the glandular organ.

Lastly: I will add, in conclusion, that, owing to the influence of the two nerves whose physiological action we have indicated, the submaxillary gland is found possessed, in reality, of an individual circulation, which, in its variations, is independent of the saliva much more fluid, and that of the great sympathetic much more viscid. This phenomenon is particularly observed when all the nerves of the gland being cut, we galvanize the ends which yet hold to the gland.

general circulation; and, what I say here for the submaxillary gland, may, without doubt, be advanced for all the organs in the economy. The pressure of the arterial system and the cardiac impulsions are common mechanical conditions that the general circulation dispenses to all the organs.

But, the special nervous system, which animates each capillary system and each organic tissue, regulates, in each part, the course of the blood in relation with the particular functional chemical states of the organs. These nervous modifications of the capillary circulation occur on the spot, and without any perturbation of the circulation being borne to the neighboring organs, and, for still stronger reason, to the general circulation. Each part is tied to the whole by the common conditions of the general circulation, and, at the same time, by means of the nervous system, each part can have a proper circulation, and individualize itself physiologically.

Such are the special physiological conditions impressed by the nerves on the capillary circulation, and which appeared to me indispensable to make known, before commencing the study of the chemical conditions of the various venous bloods. It remains, at present, to know what are the chemical modifications of the blood, which give birth in the physiological conditions we have indicated, to these alternated red and dark colorations of the glandular venous blood. This will be the subject of the following communication.

*II. On the quantity of oxygen which the venous blood of the glandular organs contain, in the states of function and repose, and on the employment of the oxide of carbon to determine the proportion of oxygen in the blood.*

In a communication made to the Academy, January 25th,* I demonstrated that, in the normal or physiological† state, the

* See No. II. of this Journal, April, 1858, p. 233.

† In the physiological condition, the excitability of the secretory nerve is always accompanied with acceleration of the circulation and a red coloration of the venous blood. These phenomena are so much the more marked, as the glandular organ is smaller and more independent in the disposition of its vessels of the circulation of the neighboring organs. I do not know any gland where the phenomenon is so visible as in the submaxillary gland of the dog, which fills all these conditions. But, so as not to be misunderstood on the subordination of venous blood of the glands is red, when these organs expel the product of their secretion, and that it is dark when the organs expel nothing and are inactive.

In another communication (see the above), I have indicated by what physiological mechanism two orders of nerves hold under their influence the variations of color which supervene in the glandular venous blood.*

To-day, I desire to examine the chemical modifications of the blood, which are in relation with its changes of coloration in the same vein.

But I must hasten to say, it is not question here of a chemical analysis of the blood. In this examination of the glandular venous blood, it will only be question of the relative quantity of the oxygen, the gas to which is always attributed the ruddy color of the blood. Besides, I would not have permitted myself this

---

these various phenomena, I will remark, that all I have said proves clearly that this coloration of the venous blood is, in consequence of the action of the nerve which accelerates the circulation, and not a cause of secretion; for it occurs after the section of the great sympathetic, without there being any secretion. So that, if one puts an obstacle to the flow of the blood in the glandular vein, and at the same time excites the secretory nerve, the secretion may still occur, although the blood, accidentally retarded in its course, cannot flow red. In certain voluminous glands, as in the parotid of the horse, the whole of the blood is renewed with more difficulty in the gland, on account of its volume, and because of the communication of the glandular veins wiih the neighboring muscular veins, which furnish an excessively dark blood, in the masticatory movements of the animal. Also, it does not appear that in this gland, the phenomenon has been discovered, although it exists, but masked by the circumstances I have indicated. In acting thus the part of cause and effect, we see that the essential physiological action of the secretory nerve is to accelerate the circulation, and render the nervous blood red, when the acceleration is as intense as possible. There is no reason to find contradictions in the less marked effects of the phenomenon which result from circumstances entirely secondary.

* Since then, I have pursued my researches on the nerves which accelerate or retard the capillary circulation, and I have found that these two orders of nerves are not to be met with alone in the glands, but that they exist in other parts of the body. I have established, principally in the dog, that the filaments of the mylo-hyoidien branch of the inferior maxillary nerve of the fifth pair accelerates the circulation in the vessels of the face. I will give, at a future time, these experiments, in occupying myself necessarily with the phenomena of local circulations, which are yet but little understood.

encroachment upon the domain of the chemists, if I were not influenced by entirely physiological conditions, as will be seen in employing a new and very simple means for determining the quantity of oxygen in the blood.

It is now about ten years since I made some experiments on the poisoning of animals by the oxide of carbon, that I have repeated since in my course at the College of France, in 1853 and 1856.* While studying the toxic action of the oxide of carbon on the living animal, I was led to find that this gas rapidly poisoned the animals, because it instantly displaced the oxygen in the blood globules, and could not afterwards be displaced by the oxygen of the air. Whence, it followed, that the blood globules, in some sort paralyzed, were rendered incapable of absorbing the oxygen, and, thereafter, circulated like inert bodies, without the power of supporting life.

If all the blood globules are attacked by a sufficient quantity of the oxide of carbon to displace all their oxygen, death is almost instantaneous, and life cannot be recalled by artificial respiration; if a part of the blood has escaped the deleterious action, death does not follow until later, etc.

In a word, I considered the highly toxic action of the oxide of carbon as a consequence of its very great affinity for the matter of the blood globules. In short, the oxide of carbon has more affinity for the blood globules than the oxygen, seeing the oxide of carbon displaces rapidly the oxygen, while the oxygen is incapable of displacing in its turn the oxide of carbon.

It is this singular property of the oxide of carbon, of which, I believe, I was the first to recognize the action, which conducted me very naturally to employ this gas in displacing the oxygen of the blood. This means offers above the old methods the advantage of being more exact, because, by the same toxic action to which the oxide of carbon subjects the blood, the causes of the disappearance of the oxygen during the operation are removed.

For two years, I have employed this method in a great num-

* Notes of M. Bernard's lectures on the blood, with an appendix by Walter F. Atlee of Philadelphia, 1854; pp. 19 to 22. *Laçons sur les effets des substances toxique et medicamenteuses.*—Paris, 1857.

ber of researches; and, last winter, at the College of France, in my course, the principle subject of which was the study of the blood, I developed publicly the advantages of this means of analysis, basing it on the numerous experiments made by M. Leconte, and which were instituted for determining the relative quantity of oxygen in the blood of the different organs of the body.

Here, in few words, is my mode of operating: I draw out the blood from the vessels by means of a graduated syringe, and pass it rapidly, by the aid of a covered iron canula, into a graduated glass tube placed over mercury, and previously containing the oxide of carbon gas. I thus obtain the blood protected from the contact of air. (*Loc cit*, p. 166). As soon as the blood is introduced, I shake it strongly, so as to effect the mixture, and prevent coagulation. I maintain the contact of the blood, add oxide for an hour or two, at a temperature of 30 to 40 degrees, being careful to agitate the blood during this period at two or three different times. The total volume of the gas does not ordinarily change, because the oxide of carbon displaces the oxygen volume for volume.* Under the influence of the oxide of carbon, one sees all kinds of blood take a persistent vermilion tint, which I signalized long ago, as characterizing the action of the oxide of carbon, as well in the blood-vessels of the living animal as upon blood treated out of the body.

I habitually employ, for each experiment, 25 cubic centimetres of the oxide of carbon for 15 cubic centimetres of blood. With this quantity of gas, all the oxygen of the blood can be displaced, which can be proven by adding a new addition of oxide of carbon, and in this second washing no appreciable quantity of oxygen is found.

To analyze the gaseous mixture in which the displaced oxygen is found, we use the methods habitually employed. The carbonic acid is treated by potassa, the oxygen by pyrogallic acid, and the oxide of carbon, when we have recourse to

* I have already mentioned this displacement volume for volume of the oxygen by the oxide of carbon (course of 1856, page 84); but I have since seen, when there is much carbonic acid, that there was augmentation of the total volume of the gas.

it, is made by its transformation into carbonic acid by the electric spark.

After these preliminaries—a little long, but, as I believe, necessary—I come to the essential object of my communication, which is, to ascertain whether the red glandular venous blood contains as much, or more, oxygen, than the dark glandular venous blood. I have thought it necessary to put the question thus. In fact, in the present state of our knowledge, we cannot make but two hypotheses on the cause of the vermilion coloration of the blood which flows from the gland, when the activity of its functions are such as we have described, animated by pulsations, as the arterial blood, when the secretion is very intense. It might be thought that the red venous blood is simply the arterial, which has traversed the capillaries with so much rapidity that it had not time to become venous—that is to say, to have its oxygen take in its place carbonic acid.

But, it may also be admitted, that the red venous blood is an ordinary venous blood, with this difference, that it does not remain dark, because, being formed at the moment of secretion, it is deprived, by the glandular excretion, of its carbonic acid, which would have rendered it dark; thus it occurs when the gland does not secrete, and the carbonic acid cannot escape.

This last opinion acquires a good degree of probability by the fact that all the liquid secretions contain a considerable proportion of carbonic acid, either in solution or combined. The comparative quantity of oxygen contained in the blood at its entrance into the gland, and its exit from the same organ, not alone capable of deciding the one or other of these two hypotheses, if, at its exit from the gland, the red venous blood contained more oxygen than the dark venous blood, and as much as the arterial, it is clear that it is not venous blood. If, on the contrary, the red venous blood gave less oxygen than the arterial, and in an equal proportion to that contained in the dark, we ought to accept the received opinion, namely: that, during the secretion, the arterial blood became venous, as usual, with this peculiarity—that it remained red; because, it was deprived at the moment of its carbonic acid, instead of later eliminating it through the pulmonary organs.

These, therefore, are the terms of the problem that I propose to solve. Let us see what the experiments really teach us.

I experimented with the blood of the renal vein, because the volume of the organ permits us to obtain with facility sufficient quantities of blood for analysis.

On a vigorous dog, during digestion, after having exposed the renal vessels of the left side with proper precautions, I withdrew rapidly, and brought immediately in contact with 25 cubic *centimetres* of oxide of carbon,* 15 cubic centimetres of blood from the renal vein. During the time, the urine was flowing abundantly from the ureters, and the venous blood was nearly as ruddy as that of the artery. Immediately afterwards, one of the numerous divisions of the renal artery, at its entrance into the kidney, was cut, and, from its central end, I withdrew 15 cubic *centimetres* of blood, which I likewise mixed with a similar quantity of oxide of carbon.

Then, to trouble the urinary secretions, I removed the fatty capsule of the kidney. The urine ceased, after a few instants, to flow by the ureter, and the blood of the vein became as dark as the venous blood of the vena cava.

At this moment, I withdrew 15 cubic *centimetres* of the dark venous blood from the renal vein, which was, like the two others, treated with 25 *centimetres* of the oxide of carbon. After remaining an hour in a bath at a temperature of from 30 to 40 degrees, the analysis of the gas in contact with the three kinds of blood above designated, gave the following results for the quantities of oxygen they contained, calculated in 100 volumes of blood:

| | Volumes of Oxygen. |
|---|---|
| 1. For the red venous blood, | 17.26. |
| 2. For the arterial blood, | 19.46. |
| 3. For the dark venous blood, | 6.40. |

In a second experiment, there was found 16 for the 100 of

---

* This rapid withdrawal of the blood from the renal vein is tolerably difficult to accomplish. We must avoid tieing the vein; for, as soon as we do, the blood becomes black from obstacle to the circulation. It is on this account that I prefer to penetrate the vein from the right, and plunge the canula of the syringe into the left renal vein, in which the circulation is not interrupted.

oxygen in the red renal venous blood; 17.44 in the aortic arterial; and 6.44 in the venous blood of the vena cava.

From these experiments it is seen, that the red venous blood of the kidney (and it is presumable that it is the same of the other glandular bloods) differs from ordinary venous blood, in that it is not—so to speak—deoxydized. Thus we find our first hypothesis verified, since this blood preserves the characteristics of the arterial. Nevertheless, if this is true of the proportions of oxygen found, the proposition is not absolutely exact. In fact, the red glandular venous blood contains much less fibrine than the arterial; it contains less water, because it has furnished that of the secretion; and, again, this red venous blood is manifestly more corruptible than the arterial blood—that is to say, becomes spontaneously dark much more rapidly when it has been withdrawn from the vessels, etc.*

Be it as it may, in directing our attention for the moment to the unique object of our present research—that is to say: to what concerns the proportion of oxygen in the glandular venous blood, we discover this very singular fact, that it is during their functions, when they are secreting, that the glands allow the blood to pass without deoxidizing it, while, when they are not acting, and do not expel any product, the blood which escapes is dark, deprived in great part of its oxygen, and charged with carbonic acid.† Here presents anew this antagonism between

* These same properties are remarked of the venous blood of the head, when the great sympathetic has been previously cut at the middle of the neck. The experiments that I have made on this subject since 1852 show, that after the section of the sympathetic, the circulation is considerably accelerated; the temperature increases; the nervous blood becomes red, and the pressure of the circulation is augmented. If we galvanize the superior or peripheral end of the sympathetic, the circulation is diminished in rapidity, the vessels contract, and the temperature is lowered at the same time that the blood becomes very dark. It is particularly in the horse that these facts present themselves with the greatest prominence. This great tendency to decomposition of the red venous blood necessitates that we operate with great celerity in putting it in contact with the oxide of carbon, which prevents its becoming venous, and its deoxidation by the formation of carbonic acid.

† I will not examine the question of the quantity of carbonic acid produced; but will only say, that, with the oxide of carbon, I have never found a quantity of carbonic acid which corresponded to the quantity of oxygen lost; which the glandular system and the muscular, to which I have often called attention. In the muscles, the blood flows so much the more dark, and the more deoxidized, as the organ acts and contracts more energetically, in the glands, the blood escapes so much the more red, and less deoxidized, as the organ acts, secretes with greater intensity. But, ought we to consider this antagonism in the phenomena apparent as a radical difference in the modes of nutrition, and the functions of the glands and the muscles? In a word, may we say, that the muscles consume the oxygen, in direct ratio to their functions of activity? It is the opposite with the glands. Or, ought we, rather, in the face of this muscular conclusion, conceive doubts of our manner of designating the functional states of the glands?

I am of this last opinion. I think that these researches will lead to another interpretation of what has been termed the states of function and repose of the glands, and will cause us to recognize a state of chemical activity, and another state of functional mechanical action. I could already bring various arguments in favor of this opinion; but, I will conclude, with the plain facts that I have above indicated, limiting myself to signalizing this obscure side of the question, which will serve as a *point de depart* for future researches.

